# Effect of Nanoparticles Surface Bonding and Aspect Ratio on Mechanical Properties of Highly Cross-Linked Epoxy Nanocomposites: Mesoscopic Simulations

**DOI:** 10.3390/ma14216637

**Published:** 2021-11-04

**Authors:** Maxim D. Malyshev, Daria V. Guseva, Valentina V. Vasilevskaya, Pavel V. Komarov

**Affiliations:** 1 Departments of Physical Chemistry and General Physics, Tver State University, Zhelyabova 33, 170100 Tver, Russia; bggf@bk.ru; 2 A.N. Nesmeyanov Institute of Organoelement Compounds RAS, Vavilova St. 28, 119991 Moscow, Russia; guseva@polly.phys.msu.ru

**Keywords:** polymer nanocomposites, mesoscale simulations, polymer networks, nanoparticles, surface modifier

## Abstract

The paper aims to study the mechanical properties of epoxy resin filled with clay nanoparticles (NPs), depending on their shapes and content on the surface of a modifying agent capable of forming covalent bonds with a polymer. The cylindrical clay nanoparticles with equal volume and different aspects ratios (disks, barrel, and stick) are addressed. The NPs’ bonding ratio with the polymer (*RGC*) is determined by the fraction of reactive groups and conversion time and varies from *RGC* = 0 (non-bonded nanoparticles) to *RGC* = 0.65 (more than half of the surface groups are linked with the polymer matrix). The performed simulations show the so-called load-bearing chains (LBCs) of chemically cross-linked monomers and modified nanoparticles to determine the mechanical properties of the simulated composites. The introduction of nanoparticles leads to the breaking of such chains, and the chemical cross-linking of NPs with the polymer matrix restores the LBCs and strengthens the composite. At small values of *RGC*, the largest value of the elastic modulus is found for systems filled with nanoparticles having the smallest surface area, and at high values of *RGC*, on the contrary, the systems containing disk-shaped particles with the largest surface area have a larger elastic modulus than the others. All calculations are performed within the framework of a mesoscopic model based on accurate mapping of the atomistic structures of the polymer matrix and nanoparticles into coarse-grained representations, which, if necessary, allow reverse data mapping and quantitative assessment of the state of the filled epoxy resin. On the other hand, the obtained data can be used to design the functional materials with specified mechanical properties based on other practically significant polymer matrices and nanofillers.

## 1. Introduction

The development of new polymeric materials remains a priority goal, especially in consumer-oriented industries, such as food, processing, and pharmaceuticals [1,2,3,4]. The constantly growing demand for new materials arouses increased interest in understanding at the molecular level the causal relationship of changes in polymer properties due to the peculiarity of the composition of the system and, in particular, the embedding of nanoparticles (NPs) used to regulate a wide range of properties [5,6,7,8,9,10]. With weight fractions comparable to traditional fillers, NPs have the potential for a much more uniform volume distribution as they have a larger surface area and allow for specially designed surface shape and functionality.

Currently, creating biodegradable and recyclable polymeric materials (BRP) has come to the foreground due to the improper disposal of used polymeric materials and the growing pressure of polymeric waste on the environment [11]. Although, in their operational characteristics, BRPs are in many respects comparable to traditional polymers, such properties as insufficient mechanical strength, low temperature of thermal destruction, and others limit the areas of their implementation. Therefore, the problem of developing green composites extends to the search for an environmentally friendly filler. It is believed that natural aluminosilicates (feldspars, clay minerals, etc.), which are biologically inert, are the most suitable for these purposes. Even a small volume fraction of clay NPs (1–5% vol.) can significantly improve the mechanical properties of polymers [12,13,14,15,16,17,18,19,20,21,22,23] in terms of thermal stability [23,24,25,26], reducing gas permeability [27,28], and flammability [29] and, in some cases, can even increase the rate of decomposition of biodegradable polymers [30,31].

The design and production of polymer/clay nanocomposites remain a challenging problem due to the large amount of work associated with laboratory searches. All the factors, such as the type of polymer matrixes, nanoparticles, surface modification, their suitable volume fractions, way of preparation, and processing conditions are considered essential. The problem is that there is no complete understanding of how interfacial interaction and the structure of a composite at the nanoscale lead to the formation of mechanical response, toughness, elasticity, glass transition temperature, and other physical properties. This fact generates significant interest in the theory and modeling related to the production of new materials. Computer simulations provide an excellent opportunity to directly study the effect of nanoparticles on the material properties at the molecular level. Simulations of polymer systems are difficult due to the necessity of accounting for the complex interaction of structural elements and the relaxation processes covering wide ranges of spatial scales and time intervals [32,33,34,35,36,37]. Layered silicates make simulations even harder [33], for several reasons. First, considering that aluminosilicate nanoplates have a thickness of 1–5 nm and a diameter of 25–500 nm [7,14,34], calculations within the framework of full-atomistic models using the actual sizes of NPs require the construction of systems containing about a billion atoms. Second, accounting for such dimensions in full is possible only within the framework of the finite element method. However, in this case, information about the features of the structural organization of the polymer matrix is lost. To date, simulations of layered silicates by full atomistic methods (molecular dynamics and Monte Carlo) have been implemented for relatively small systems [35,36,38,39,40,41,42,43].

Using mesoscale (coarse-grained) modeling to examine the structural properties of polymers filled with layered silicates is a good choice if we want to maintain the polymer matrix’s key structural details. The main idea of mesoscale modeling is to solve a compromise problem of preserving the main features of the structure and dynamics of the original system with the maximum possible decrease in the number of degrees of freedom. The transition from atomistic to coarse-grained representation is known as mapping, and the back transition is known as reverse mapping [32,37,44], wherein one strives to preserve the structural features and properties (the diffusion coefficient, the radius of gyration, etc., [35]) of large molecules such as polymers. To build coarse-grained models, individual groups of atoms and sometimes statistical segments of polymer chains are modeled as beads connected by springs. The springs are a model of how the beads interact with each other. Mesoscale models can be used within the coarse-grained molecular dynamics method [37,45] if there is sufficient information about the interaction of all the beads. Certain methods, such as the Brownian dynamics [35,46] and dissipative particle dynamics [47,48,49,50], complement the coarse-grained molecular dynamics simulation. These methods add dissipative, random, and/or repulsive forces between the model particles to efficiently model the materials under study. Due to the reduction in the number of degrees of freedom in these methods, it is possible to simulate systems (while preserving the key structural elements) whose dimensions are difficult to access for atomistic modeling [51,52]. Results obtained with coarse-grained models can be directly compared with the data from real-life experiments [35,51,52].

Composites containing silicate layers have already been intensively studied within the framework of dissipative particles dynamics (DPD) [53,54,55,56,57,58,59] to reveal their mutual ordering and structural properties [53], including taking into account the cross-linking reaction [54]. DPD is most often used in multiscale modeling [55,56,57,58,59] to capture the density profiles of polymers relative to the clay nanoparticles, which is necessary (in some studies) for the parameterization of continuous methods.

At the same time, it is worth noting a number of problems and limitations of coarse-grained modeling. When constructing coarse-grained models, it is necessary to take into account which physical property is to be studied. For example, in many engineering applications, it is essential to predict the main trends in stress–strain and Young’s modulus, which describe the observed behavior of the material under study in the region of a lower-scale continuum. In this case, the construction of the model will consist in finding such a scale level with smaller linear dimensions so that the model can simulate the behavior of a heterogeneous system as accurately as possible. To choose the optimal size of the simulation cell, it is also necessary to consider which boundary conditions are used as the latter can affect the results obtained [37]. It has been shown that periodic or mixed boundary conditions provide the studied object with a more realistic predictable response [60,61]. In many cases, the smallest possible repeatable structure size is selected as the minimum cell size for a material with a regular structure. For simulating amorphous substances and materials, the minimum cell size is a size that statistically represents the system structure. However, in general, there is no consensus or definitive recommendation on the minimum size of a simulation cell that will provide acceptable accuracy. Usually, the larger the cell size of the simulation, the higher the accuracy of the calculations [37]. Therefore, in the case of amorphous materials, the convergence in this parameter must be checked separately. It is most challenging for network polymers due to the necessity of checking the preservation of the principal topological invariants of the polymer network [62,63].

In this paper, we present a mesoscale model of a highly cross-linked (network) polymer melt filled with nanoparticles. For its implementation, the method of dissipative particle dynamics is used. We focus on identifying the causes responsible for the enhancement of the mechanical properties of nanocomposites. In our model, which further develops the computational scheme [63], we also assume that the NP surface is coated with a modifier that can be bonded with a polymer matrix (i.e., with an anchor agent). We use cylindrical nanoparticles with different ratios of diameter and height (aspect ratios) to study the effect of shape.

Next, we organize the paper as follows: Section 2 describes the material model and the main details of the calculation scheme. In Section 3, we discuss the results obtained and how they relate to real systems. Finally, in Section 4, we summarize the results of our research.

## 2. Model and Parameterization

All the calculations were performed with the dissipative particle dynamics, first proposed by Hoogerbrugge and Koelman [47,48,49,50]. As this methodology has been well described, for example in the works [53,54,55,56,57,58,59,63,64,65,66], we will not dwell on it in detail.

A cross-linked polymer based on diglycidyl ether of bisphenol-A epoxy resin (DGEBA) and tricarboxylic fatty acid (FAT3) was chosen as a prototype of the polymer matrix. This polymer combines the advantages of thermoplastics and thermosets and belongs to a new type of high molecular weight compounds called vitrimers, first investigated by the group of prof. Leibler [67]. Vitrimers contain dynamic bonds that can rearrange when the temperature rises. Due to this, products based on them can soften when heated. This makes it possible to give them a new shape that can be fixed when cooled and to weld their parts.

To build a model of the nanocomposite, we use the reaction version of DPD, based on the concept of mesoscale chemistry [62,63]. This concept implies that as a result of the mapping of the initial chemical structures to a coarse-grained representation, not only their structural features are preserved, but all the reactive groups. To build equivalent models, we use the so-called composite beads constructed from the ordinary DPD-beads (hereinafter, we will call them simply “beads”) and linkers [62]. Beads are structureless spherical particles (with diameter σ = 1 and mass *m* = 1) assigned to fragments of the initial molecular structure. In addition, we assume that *k*_B_*T* = 1 and τ *_t_* = σ(*m*/*k*_B_*T*)^1/2^ (*k*_B_ is the Boltzmann constant, *T* is the temperature), which gives the dimensionless system of units. Each fragment (subsystem) is assigned beads of its type. Each type of bead carries information about the features of the intermolecular interaction of the corresponding fragment. Large molecules can be represented by several beads connected by springs. It is assumed that beads can additionally participate in other interactions such as the deformation of bond angles, etc., (see Refs. [63,68]) in order to consider the system’s spatial structure.

Linkers are identified with the reactive groups (RG) involved in the chemical reactions. Unlike DPD beads, linkers can form new covalent bonds and participate only in interactions that describe the deformations of bonds and the angles between them. If the molecular fragment does not contain reactive groups, the linkers are not attached to it. The reactivity of each linker is determined by the reactivity of the corresponding atomistic groups, which in our model are regulated by the probability *w_ij_*. The indices *i* and *j* correspond to the types of beads to which the linkers are attached. Moreover, each linker has a valence mark of 0 or 1, which means its prohibition or allowance, respectively, to react.

The use of composite beads has a significant advantage. Such a representation makes it possible to perform the reverse mapping procedure (transition back to the atomistic model) much more accurately. In addition, bonds and angles between beads and linkers control the equivalence of coarse-grained and atomistic structures. The Hooke potential with a stiffness constant *K* ^(bond)^ = 200 describes the deformation of all bonds in the composite beads. This and other quantities are given in dimensionless DPD units. To control the relative position of the linkers, the monomer model also uses the harmonic deformation potentials of angles with a stiffness constant *K*^(angle)^ = 2. The equilibrium value of each angle is determined by the mutual position of the reaction groups relative to the centers of mass of the initial structures.

**Figure 1:**
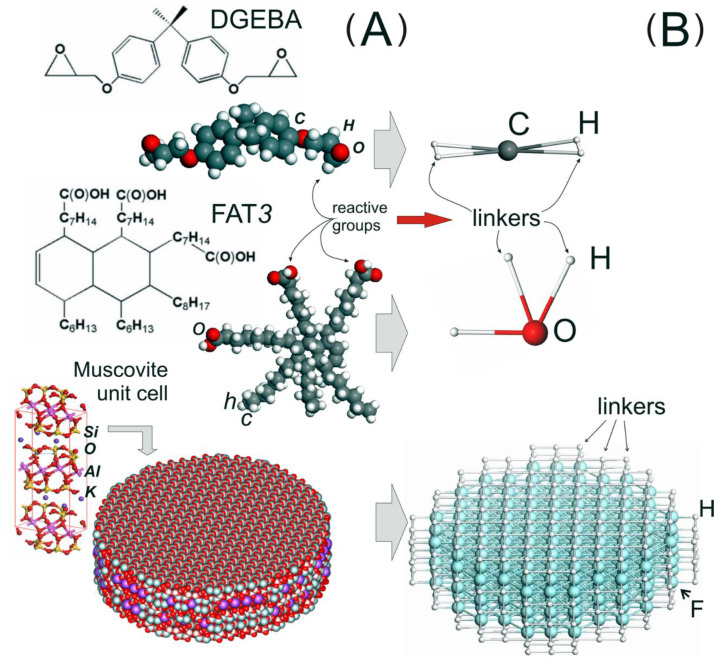
(**A**) Chemical structures and atomistic models of epoxy resin: diglycidyl ether of bisphenol-A (DGEBA), hardener: tricarboxylic fatty acids (FAT3) and muscovite (H, C, O, Si, K, and Al are the names of chemical elements). (**B**) The corresponding coarse-grained models. We use composite beads, which consist of conventional DPD beads and semi-virtual particles, called linkers, to simulate chemical reactions at the mesoscale level. When we are mapping atomistic structures onto coarse-grained representation, the linkers with the corresponding valence are situated at positions of the reactive atoms. The DPD beads can also be cross-linked to each other to imitate the shape of molecular structures. DPD beads are involved in all types of interparticle interactions. Linkers participate in intramolecular interactions when the bonds and bond angles are deformed. In addition, they can form new covalent bonds. In this part of the figure, C, O, F depict DPD beads and H linkers.

The designed model includes subsystems of three types. They correspond to the comonomers DGEBA and FAT3 and the fragments of clay NPs, which are designated by the symbols C, O, and F, respectively; see Figure 1. The symbols of the chemical elements are used as types of specific beads solely for visualization convenience. Figure 1 shows the transition from atomistic structures (Figure 1A) to their equivalent coarse-grained representation (Figure 1B). For the monomers DGEBA and FAT3, we built models from one bead with four and three linkers, respectively. In the figure, the linkers are designated as particles of type H. The use of such a simplified representation is dictated by a compromise in comparing the radii of gyration of the model components and preserving the reactive degree of freedom of the monomers. In the selected view, we can control the topology of the polymer network. Thus, in our model, the average size of comonomers determines the scale unit of the system σ ≈ 20 Å.

The choice of representation for the monomers of the matrix dictates the way of the nanoparticle construction. The clay nanoparticle model was built as a set of connected DPD beads (see Figure 1B), making it possible to reproduce the shape of the NP. Due to this, they can influence the local structure of the polymer matrix and participate in the transfer of mechanical load through the simulation cell. Based on the notion that exfoliated clay particles are thin plates [7,14] and for reasons of simplicity, for the coarse-grained NP model we chose a disk shape with a diameter *D* = 7.04 σ (11 beads) and a height *H* = 1.92 σ (3 beads). It was cut from a three-dimensional array of type F beads on a cubic lattice with an edge length of 0.64 σ. Fulfillment of this condition makes it possible to achieve a spatial packing density of type F beads equal to 3 σ ^–3^, which prevents the penetration of the polymer into the filler. We have introduced two types of bonds between the F beads. The first type has an equilibrium length of 0.64 σ; they form the faces of a cubic lattice. The second type, with an equilibrium length of 0.905 σ, corresponds to the diagonals between the beads of each face. The presence of these bonds gives the NPs rigidity, which ensures the preservation of their shape.

In our model, we assume that the NPs have a modified surface. Usually, the modification is carried out using special compatibilizers to create compatibility between the hydrophobic polymer matrix and the hydrophilic clay NPs. Rogers et al. [69], using contact angle measurements, have found that the organophilic coating of clay particles can improve polymer-clay wetting. It has been experimentally demonstrated that the use of organophilic surface modifiers makes it possible to realize the uniform dispersion of the NPs [70] and better adhesion of the polymer, which has a positive effect on the mechanical properties of the system [14,15,16,17,71,72,73,74]. In some cases, anchoring coupling agents which afford two types of reactivity (with inorganic and organic compounds) can improve the wettability at the interface between the inorganic and organic materials through the cross-linking or coupling of two dissimilar materials [9,75,76,77].

To account for the surface modification, linkers were attached to each bead on the NP surface to simulate the modifier. They were located at the nodes of the cubic lattice adjacent to the surface beads. Considering that the reactive groups of the anchor agent can be located on oligomeric chains [9,75,76], between the F beads and the linkers, we introduced only the bonds that correspond to the faces of the cubic lattice (see Figure 1). We assume that the linkers on the NP surface can react with epoxy resin monomers (type C bead). By changing the relative fraction of linkers that are “*allowed*” to form new bonds, *f*_l_, and the time of simulating the polymerization reaction, we can vary the reactive group conversion (*RGC*). This characteristic is defined as the ratio *RGC* = *n_r_*/*N_l_* (*N_l_* is the number of linkers on the NP surface, *n_r_* is the number of reacted linkers) and is related to the number of bonded reactive groups on the surface, which determine the degree of cross-linking of NPs with the matrix. The *f*_l_ parameter ranges from 0 (linkers are chemically neutral) to 1 (all linkers can react). The linker valence is assigned randomly when the system is created. The *RGC* value was calculated upon completion of the cross-linking reaction. The Hooke potential with the same stiffness constant *K*^(bond)^ = 200 (as in the case of composite beads) describes the deformation of all the bonds in the NPs. Note that the chosen way of constructing NPs allows us to consider and account for many of the factors responsible for transferring the load through the bulk of the material. These include the weight fraction of the NPs, their shape, the degree of dispersion in the volume, mutual orientation, and the chemical characteristics of the surface.

**Figure 2:**
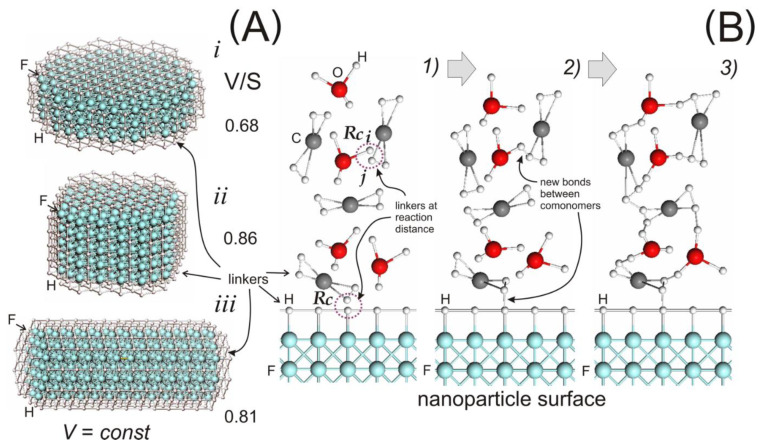
(**A**) Constructed nanoparticles of three types with different aspect ratios. (**B**) Curing reaction: (1) two linkers at a distance < *R*_c_, (2) new bonds formation between DGEBA-FAT3 and DGEBA-NP, (3) a fragment of the polymer network formed during the chemical reactions. All colors in this figure are the same as in Figure 1. The letters C, O, and F depict DPD beads and H linkers.

To test the influence of the NP shape on the mechanical properties, we constructed additional types of NPs in the form of “barrel” and “stick” (Figure 2A), also covered with linkers. Thus, we built three types of NP discs: (type *i*), barrel (*ii*), and stick (*iii*). When constructing, we kept the NP volume constant (*V* = const). Thus, the NPs differed only in the aspect ratio, which could otherwise be characterized by the ratio of their volume *V* to the surface area S (excluding linkers). It is worth noting that, of course, most clays in a layered state produce plate-like NPs. Nevertheless, NPs of a mineral such as halloysite [78] are nanotubes formed by a layer of rolled silicate. Thus, we can conditionally identify the first and third types with the NPs of clays of various shapes. Nanoparticles of the second type with the highest *V*/*S* value (smallest surface area) are considered reference particles.

Let us dwell on the features of modeling the cross-linking reaction of DGEBA and the FAT3 monomers and DGEBA with the NPs. If two linkers, allowed to react, approach each other at a distance less than the cut-off radius *R*_c_ = 1 σ (see. Figure 2B), a random number is generated. If it is less than *w*_ij_, a new bond connects these linkers, and their valence is set to zero. As in [63], we use the probabilities *w*_CC_ = 0.001 and *w*_CO_ = 0.0025. For the reaction of DGEBA with the NPs, we chose *w*_CF_ = 0.001. The rest of the *w*_ij_ values are equal to zero. Note that we do not consider the reaction of the associative exchange of intermonomer bonds (intrinsic to the vitrimers) in this work.

To determine the miscibility of the subsystems in our model, we also set the parameters of the maximum repulsion of the beads *a_ij_*. These parameters are related to the intermolecular interaction forces and are expressed through the Flory–Huggins parameters χ*_ij_* [79,80]. If 0 ≤ χ*_ij_* < 0.5 (25 ≤ *a_ij_* < 26.7) the beads mix well. At large values of *a_ij_*, the corresponding subsystems are separated. In [63], the estimate χ_OC_ = 0.11 was made, and the miscibility of the DGEBA, FAT3, and silica NPs was evaluated. For the pairs DGEBA-SiO_2_ and FAT3-SiO_2_, the value χ = 1.4 was obtained, which, as the surface of clay NPs is often a layer of silicon dioxide in the case of unmodified NPs, gives *a*_DGEBA-SiO2_ = *a*_FAT3-SiO2_ = 30. This indicates the poor compatibility of such NPs with the matrix and the need to modify the NPs’ surface. In the present paper, we use the work results of [81] in which the compatibility of epoxy monomers 3,4-epoxycyclohexylmethyl-3,4-epoxycyclohexanecarboxylate with silicon dioxide NPs modified with the anhydride of 3-(triethoxysilyl)-propyl-succinic acid was assessed. For them, a negative value for the χ parameter was obtained, which indicates their good miscibility. Additionally, the miscibility of the various surface modifiers with the comonomers DGEBA and FAT3 was evaluated; see Table S1 in Section S1 (“S” denotes a reference to the supporting information). Taking into account these estimations, we chose *a*_OC_ = 25.4 and *a*_CF_ = *a*_OF_ = 25 as repulsion parameters.

## 3. Methodology of Simulations

### 3.1. Systems Preparation

We prepared coarse-grained models of nanocomposites containing 16 NPs of three types with different aspect ratios (shown in Figure 2A) and an unfilled polymer network as a reference system. All the model parameters are described in the previous section. As we focused on the study of the effect of the cross-linking degree of NPs with a polymer matrix, the number of NPs was fixed. We did not consider the problem of NP aggregation and, on the contrary, tried to obtain a uniform distribution of NPs in the cell volume. We also assume that the surface modifier has a chemical nature similar to that of the polymer matrix, which is set by the choice of the parameters of the repulsion of beads *a_ij_*, described above.

The initial distribution and the orientation of the NPs were generated randomly in a cubic cell with periodic boundary conditions with an edge length *L* = 17 σ. Taking into account the selected value of the scale unit σ, the chosen size corresponds to ≈340 Å. The number of monomers of the polymer matrix was equal to 3ρ^−3^, which corresponds to the density of the system in the DPD models [48,50]. The following ratio of the volume fractions of DGEBA monomers was chosen: DGEBA: FAT3 = 5/7:2/7 to obtain a polymer network with the maximum degree of cross-linking. The construction and the equilibration of the systems were carried out using the reaction version of DPD, adapted to the objectives of this study [82]. To integrate the system of equations of motion, we use the modified Verlet method with a step of 0.002 τ*_t_* (one DPD step). A protocol of the three stages described below was used to bond the polymer matrix and the NPs.

In the first phase, we performed the initial equilibration of the systems during 200,000 DPD steps, first under NVT conditions and then under NPT ensemble conditions. The temperature and pressure of the system reached an equilibrium value, and all the monomers of the matrix were expelled from the bulk of the NPs. We monitored the distribution of the NPs using visual control and calculating the radial distribution function *g*(*r*) of their centers of mass. As seen from Figure S1, the nanoparticles uniformly filled the volume of the cell. The formation of large aggregates was not observed, although individual snapshots showed the convergence of individual NPs with each other. To reveal the orientational orderings, we calculated the orientational order parameter of the cylinder axes using the value of the second Legendre polynomial *O_p_* = (3cos θ − 1)/2, where θ is an angle between the axes of the cylinders. The results are shown in Figure S2. Overall, all the obtained values for *O_p_* were close to zero.

At the second stage, the curing reaction of the polymer matrix and its cross-linking with a modifier on the NP surface were modeled. In the case of the nanocomposites, the reaction was carried out for various values of the parameter *f_l_*, which controls the number of active linkers (with valence 1). The computational procedure for the implementation of chemical reactions was launched every 1000 DPD steps. This time was sufficient for the relaxation of the polymer network (of the new bonds and forming subchains) on a local scale. In this case, the corresponding concentration of the reagent remained approximately unchanged. The progress of the chemical reaction was controlled by calculating the degree of conversion (*DC*) and the relative number of linkers that reacted. Figure S3 shows how this quantity changes over time for the unfilled polymer and nanocomposites. The total implementation time for this stage was 3,000,000 DPD steps. As one can see from Figure S3, *DC* growth stops upon reaching 500,000 DPD steps. The maximum *DC* degree attained for unfilled polymer was 0.69. In the case of systems with NPs for which the parameter *f_l_* = 1, the maximum conversion rate was slightly higher than that of an unfilled matrix and amounted to 0.71. Note that, depending on *f_l_*, the number of reacted linkers in the NPs (characterized by *RGC*) varies from 0 to 0.65. During this phase, we observed a slight decrease in the volume of the systems. In the case of the nanocomposites, the relative decrease in the edges of the cell was 2.5%, in the case of an unfilled matrix, 3%. This behavior (known as shrinkage) is characteristic of epoxy resins. It is automatically taken into account in our model through the use of the NPT ensemble.

At the final third stage, we performed the final equilibration during 200,000 DPD steps. Figure S1B shows snapshots of the prepared composites.

### 3.2. Stress–Strain Response Calculation

The prepared systems were used to calculate the strain–stress curves for the affine (*V* = const) uniaxial deformation of the system in the NVT ensemble. This imposes several relationships on the edge lengths of the modeling cell. The change in the initial edge length *L*_α0_ is given by the expression *L*_α_ = λ_α_ *L*_α0_ (α = *x*, *y*, *z* is the direction along which deformation occurs). The deformation coefficient λ_α_ is related to the engineering deformation coefficient: e_α_ = (*L*_α_ − *L*_α0_)/*L*_α0_ = λ_α_ − 1. Additionally, we have λ_x_ × λ_y_ × λ_z_ = 1 и λ_α_^1/2^ = λ_β_ = λ_γ_ (α ≠β≠ γ).

The calculation of the true stress *t*_α_ was carried out in three stages according to the methods from Refs. [63,65,66]. At the first stage, λ_α_ was smoothly changed by the step Δλ. At the second stage, the relaxation of the system was performed, followed by averaging the components of the pressure tensor <*p*_αβ_> at the third stage of the calculation scheme and calculating *t*_α_ = <*p*_αα_> − (<*p*_ββ_> + <*p*_γγ_>)/2. The duration of each stage was chosen as equal to 200,000 DPD steps, which is sufficient to bring the system into equilibrium and a good reproducibility of results. Young’s modulus *E*_α_ was estimated from the slope of the curve *t*_α_(λ_α_) in the vicinity of λ_α_ ≈ 1, within the linear region (elastic deformation) at low tension (λ ∈ [1, 1.05]) and low compression of the system (λ ∈ [0.95, 1]), respectively.

### 3.3. Topological Analysis

To check the correctness of the construction of the cross-linked systems, we performed a topological analysis of the obtained networks. In general, the network topology is an important criterion when choosing the size of the simulation cell. The fact is that the polymer networks obtained by polymerization or polycondensation reactions are highly heterogeneous due to the stochastic nature of their formation. This makes it impossible to synthesize completely identical samples of cross-linked polymers. The larger the size of the system, the more it may contain structural heterogeneities. According to the conclusions [62], if the polymer network has insufficient volume, in the case of epoxy resins, it is less than (150Å)^3^; such a system cannot be considered suitable for predicting physical properties.

The peculiarities of the chemical structure of polymer networks determine their topological structure. Therefore, when carrying out computer modeling, it is necessary to construct samples whose structure will reflect the main topological features of the systems under study. This can be achieved by calculating and comparing their topological invariants. The structure of polymer networks can be characterized using the approach proposed by Khalatur [62] (and also applied in Refs. [63,83]). In the analysis, polymer networks are considered identical sites (vertices) connected by bidirectional edges. This allows them to be viewed as undirected graphs. In highly cross-linked systems, topological structures such as “*simple cycles*” [84] play a key role in the formation of the mechanical response of the system [63]. A simple cycle is defined as a ring with the shortest topological distances between the vertices. As we use periodic boundary conditions, a special type can be distinguished among simple cycles. Indeed, if we move along a cycle in the 3D periodic system, we can reach both the same vertex (from where we started our way), and a vertex that does not lie in the original image. Such cycles are called load-bearing chains (LBCs) and can be thought of as chains that go through the entire volume of the simulation cell and connect through periodic boundary conditions. In the case of highly cross-linked polymer networks, the LBCs are mainly responsible for transferring the load through the volume of the simulation cell. The more LBCs in the system, the more load that must be applied to deform it.

The topological analysis of the constructed networks was carried out using the techniques from the Refs. [62,63].

## 4. Results and Discussion

### 4.1. Simple Cycles and Load-Bearing Chains

**Figure 3:**
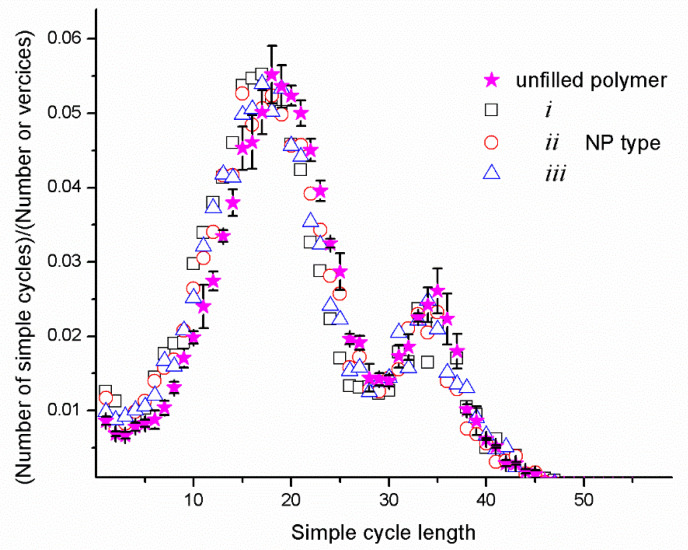
The normalized distributions of the length of the simple cycles for the unfilled network and networks containing nanoparticles of *i*, *ii*, and *iii* types (*RGC* = 0).

The topological analysis reveals that in all the systems, the simple cycles are the dominant topological structures. The fraction of simple cycles is ≈0.91, and the rest of fraction, ≈0.09, includes the sol fraction and “*trees*” [83,84]. Figure 3 shows a comparison of the length distributions of simple cycles for an unfilled polymer and the nanocomposites with the NPs to the matrix. All the distributions have well-defined bimodal profiles typical of epoxy networks [62,63]. This confirms the correct choice of the volume of the simulated system. According to Khalatur’s finding [62], the position of the first maximum corresponds to the average length of the non-periodic cycles, and the second maximum characterizes the average length of the LBCs, which are the largest cycles in the system. In the case of too-small cells, the positions of these maxima overlap, which leads to a unimodal distribution.

The profile similarity in Figure 3 indicates the same topological structure of the networks. In the case of the filled systems, the first maximum shifts towards lower values. It happens because NPs occupy part of the cell volume, which reduces the average length of the short cycles. For all the systems, the positions of the second maximum coincide well, which means that the average lengths of the LBCs are approximately the same. The nanoparticles introduce only a perturbation that changes the local structure of the matrix. The amplitude of the second maximum for the nanocomposites is somewhat smaller than for an unfilled network. This indicates a lower amount of LBCs in the nanocomposites in which the NPs play the role of obstacles to forming periodic structures. The decrease in the degree of matrix crosslinking from the introduction of nanoparticles is noted in Ref. [70]. Therefore, from the point of view of the topological structure, one can say that the filled network will be less rigid compared to the unfilled one. It should be noted that topological analysis reveals the features of the polymer network structure and does not consider the features of the polymer/NP interface. In the case of the strong adhesion of the polymer matrix, which is not detected by the topological analysis, the interface properties can significantly affect the enhancement of the mechanical properties [18,19,85,86,87]. In the publication [18], it is noted that the stretching effect of NPs occurs due to the good interfacial bonding between the NPs and the polymer matrix. This effect is especially evident in multicomponent mixtures [19].

The number of LBCs in the constructed systems was calculated relative to all three faces of the simulation cell and normalized to their area. Table 1 contains the densities of the LBCs *n*_α_ (α = *x*, *y*, *z*) in the systems used to plot Figure 3, i.e., the data for the unfilled system and nanocomposites with *RGC* = 0. The density *n*_α_ is defined as the ratio of the minimum number of LBCs passing through the planes secant to the cell perpendicular to the directions of the α-axis to the area of the corresponding secant face *S* _βγ_ (α ≠β и α ≠ γ). As can be seen from Table 1, in all the systems, the *n*_α_ values for the *x*, *y,* and *z* directions are similar within the error, which means that all the systems have a homogeneous structure. At the same time, the mean density of the LBCs <*n*> (averaged over three directions) for the unfilled systems is approximately 1.7 times larger than for the nanocomposites. This is in agreement with a decrease in the amplitude of the second maximum in the length distributions of the simple cycles for the nanocomposites with *RGC* = 0. In addition, in the nanocomposites, *n*_α_ has a larger scatter of values than in an unfilled system. This is due to the perturbation of the nanoparticles brought into the network topology. The value of <*n*> in the case of the NPs of the second type is greater than in the case of the NPs with a large aspect ratio. The fact that the NPs of the second type are more compact (they have the smallest surface area) makes it possible to create more load-bearing chains [63].

**Figure 4:**
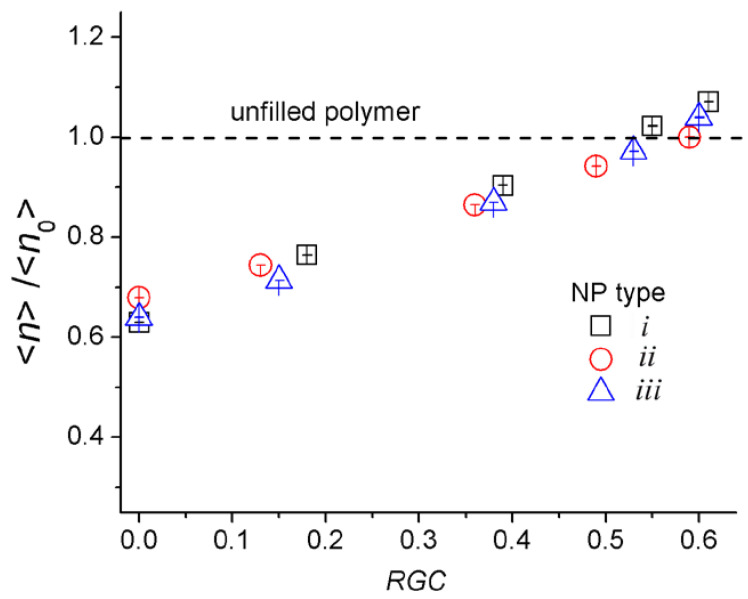
Normalized averaged density of load-bearing chains <*n*>/<*n*_0_>. Here <*n*> is average *n*_α_ (α = *x*, *y*, *z*) for the nanocomposites with nanoparticles of *i*, *ii*, and *iii* types. Reference value <*n*_0_> = 1.67 ± 0.03 is the averaged *n*_α_ for the unfilled network. The dashed line corresponds to the unfilled polymer.

Figure 4 shows the change of <*n*> for various values of the reactive group conversion. As can be seen from Figure 4, there is a gradual linear increase in <*n*> as the *RGC* increases. For the different types of nanoparticles, approximately the same trends are traced. If the *RGC* < 0.2, the average density of the LBCs in the case of the NPs of the second type (with a smaller surface area) is slightly higher than for the NPs of the first and third types (which have a higher surface area). It means that when the number of cross-links between the NPs and a polymer matrix is not large, they still play the role of the obstacle to the formation of the load-bearing chains. In this case, NPs with a smaller surface area introduce less perturbation into the matrix structure, forming a larger number of LBCs. When the *RGC* > 0.2, the situation is changed. The modified nanoparticles with a larger surface area form a larger number of cross-links with the polymer, which contributes to the forming of a larger number of LBCs. The obtained dependencies illustrate (1) the effect of the NPs’ shape and (2) the role of NPs as additional cross-linking centers.

Although the topological analysis clearly shows the mechanism for enhancing the mechanical properties in filled polymer matrices, it does not answer how significant the changes will be. As follows from Figure 4, the number of LBCs in the nanocomposites becomes more prominent than in an unfilled polymer at sufficiently large values of the degrees of cross-linking with the matrix when *RGC* > 0.6. Detailed information on the mechanical properties can be provided by direct calculations of the elastic modulus, which are discussed below.

### 4.2. Mechanical Response

**Figure 5:**
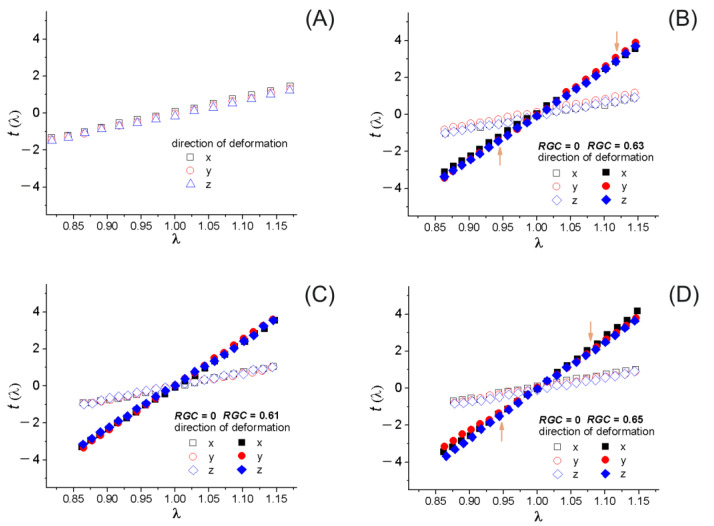
Stress–strain curves for the unfilled polymer network (**A**); the nanocomposite with nanoparticles of *i* (**B**), *ii* (**C**), and *iii* (**D**) types. Arrows in (**B**,**D**) indicate the onset of nonlinear behavior of *t*_α_(λ_α_).

To study the mechanical properties, we calculated the stress–strain dependences for the tension (λ >1) and compression modes (λ >1) in the directions of the *x*, *y*, and *z* axes using the method of Refs. [65,66]. Examples of dependencies of the real stress *t*_α_ on the deformation coefficient λ_α_ (α = *x*, *y*, *z*) are presented in Figure 5. As we were mainly interested in calculating Young’s modulus, we show in close-up the region of the corresponding elastic deformation. As can be seen, for all cases at λ_α_ ≈ 1 we observe a well-pronounced linear behavior for the functions *t*_α_(λ_α_). In the case of the systems with non-bonded NPs, the slope of the linear part of these functions is slightly less than in the case of an unfilled polymer. This indicates a weakening of the polymer’s Young’s modulus, which we associate with a decrease in the number of LBCs. For samples containing NPs of the first and third types, when the deformation exceeds a value of the order of 10%, the nonlinear behavior of *t*_α_(λ_α_) begins. In the region of small deformations, the values of *t*_x_, *t*_y_, and *t*_z_ coincide well with each other within the error limits for the tension and compression modes. This indicates the isotropic structure of the constructed systems, which is seen from the topological analysis. There is also a divergence of the *t*_α_(λ_α_) curves at the maximum and minimum λ_α_ values (shown in Figure 5) for the systems with NPs of the first and third types. It can be explained by the structural anisotropy caused by the introduction of the NPs, which is also confirmed by the topological analysis. Figure 5 also shows the reinforcing role of the nanoparticles. At large values of *RGC*, the slope *t*_α_(λ_α_) changes drastically. This indicates that the systems with bonded NPs should have a significantly higher elastic modulus.

**Figure 6:**
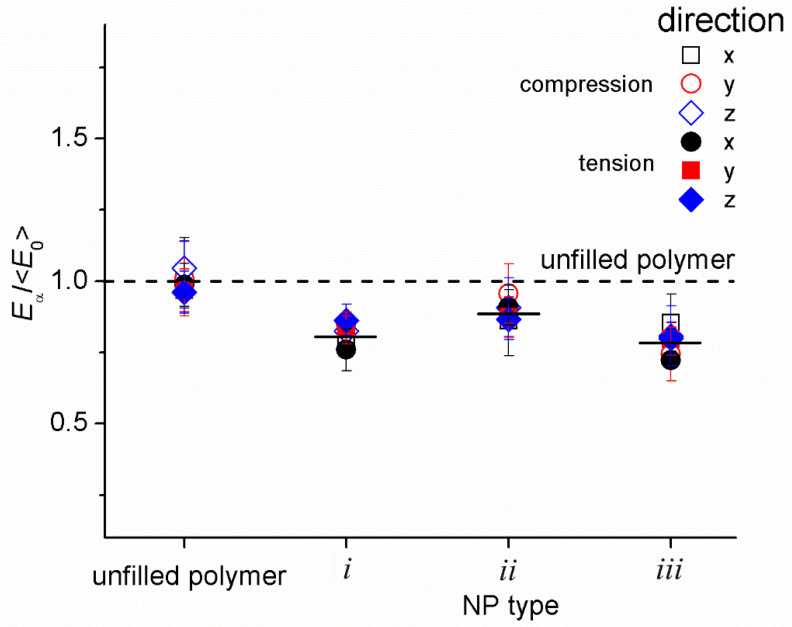
Normalized Young’s modulus *E*_α_/<*E_0_*> (α = *x*, *y*, *z*) for the nanocomposite nanoparticles of *i*, *ii*, and *iii* types. We use the average value for the elastic moduli *E*_α_ of the unfilled polymer as a reference value <*E*_0_> = 8.04 ± 0.27 DPD units. The dashed line corresponds to the unfilled polymer, the solid lines correspond to averaged values of the corresponding modulus.

Figure 6 shows a comparison of the normalized elastic moduli *E*_α0_/<*E*_α0_> for an unfilled polymer and for the nanocomposites with non-bonded NPs. The average value of the elastic moduli for an unfilled polymer <*E*_α0_> (averaged over the directions of the *x*, *y*, *z* axes and over the compression and tension modes) for the compression and tension modes was used as a normalizing factor. On the whole, the picture obtained is in good agreement with the conclusions of the topological analysis. As expected, within the error interval, *E*_x_ ≈ *E*_y_ ≈ *E*_z_ for both the compression and tension modes. Filled systems are softer than unfilled systems. The normalized modulus of elasticity for the system with NPs of the second type is noticeably higher (≈0.89), than for the systems with NPs with a large aspect ratio (≈0.8), which also can be explained by the lower number of LBCs in the latter systems.

Overall, the results obtained are consistent with the conclusion of [63] that NPs non-bonded with polymer reduce the number of load-bearing chains. In this case, the NPs play the role of a sol fraction and participate in load transfer only through intermolecular forces. Because in DPD the intermolecular interaction is implemented using soft repulsive potentials, non-bonded NPs do not have a reinforcing effect. In addition, as non-bonded NPs prevent the formation of load-bearing chains, they weaken the mechanical response.

The decrease in the elastic modulus upon the introduction of unmodified clay NPs is noted in some experimental studies [88,89,90] and a coarse-grained molecular dynamics simulation [91]. In particular, in [89] for a nanocomposite based on an ethylene-vinyl acetate (EVA) and 3 wt.% natural Na^+^ montmorillonite (MMT), the reduction of tensile Young’s modulus on about 5% was observed, compared to the neat EVA system (normalized modulus is ≈0.95). In [88], for cross-linked polyester–clay nanocomposites (Cloisite 30B-natural montmorillonite modified with methyl tallow bis-2-hydroxyethyl quaternary ammonium chloride was used as the filler) upon addition of 1 wt.%, 2.5 wt.%, and 5 wt.% of clay, the tensile modulus decreased on about 20%, 79%, and 68% (which corresponds to normalized modulus equal to ≈0.8, ≈0.22, and ≈0.32, respectively). The coarse-grained molecular dynamics simulations of the model system (of MXene-epoxy nanocomposites) with non-bonded NPs also demonstrated the decrease of Young’s modulus upon the addition of ≈1.25% of filler (on ≈5%, the normalized modulus is ≈0.95). Thus, the normalized Young’s modulus obtained in the present study is quantitatively comparable with these reported in the experimental and simulation data. This is typical of nanocomposites in which poor adhesion of the matrix to the NP surface is realized.

**Figure 7:**
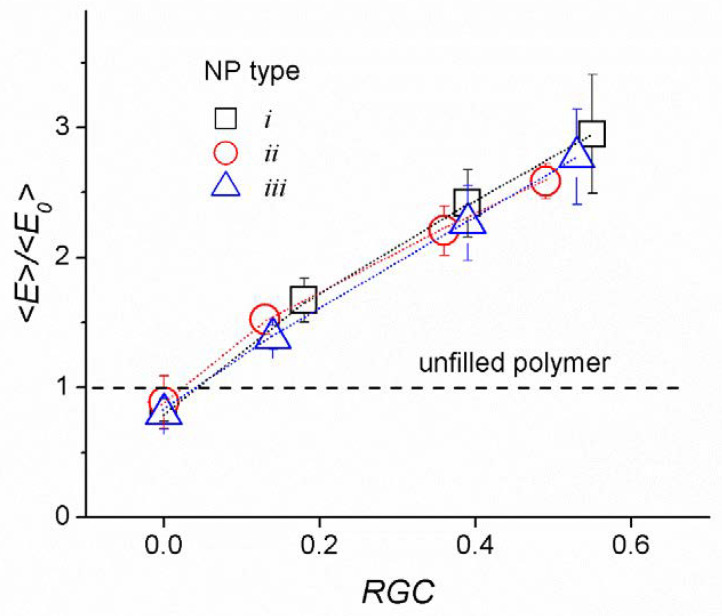
Normalized averaged Young’s modulus <*E*>/<*E*_0_> (α = *x*, *y*, *z*) for the nanocomposite nanoparticles of *i*, *ii,* and *iii* types. Here <*E*> is average *E*_α_ at different cross-linked degrees of NPs with matrices. We use the average value for the elastic moduli Eα of the unfilled polymer as a reference value <*E*_0_> = 8.04 ± 0.27 DPD units. The dashed line corresponds to the unfilled polymer.

Figure 7 shows normalized Young’s modulus <*E*_α_> on <*E*_α0_> for the NPs bonded with a polymer matrix. The values <*E*_α_> are averaged over the directions of the *x*, *y*, *z* axes for the compression/tension modes and normalized to a similar value for an unfilled system. An increase in Young’s moduli with an increase in the degree of cross-linking of the NPs with a polymer matrix is clearly seen (the value of the normalized modulus <*E*_α_>/<*E*_α0_> is equal to ≈ 1.5, 2.4, ≈3 for *RGC* ≈ 0.2, 0.4, and 0.6, respectively). This correlates well with an increase in the density of LBCs in the systems (see Figure 4). A significant improvement in the modulus of elasticity of the surface-modified clay silicates is also described in many experimental works [9,13,20,21,22,23,25,92,93] and computer simulations [65,91], where it is shown that modifiers improve the compatibility of the NPs with an epoxy matrix.

The results obtained for *RGC* ≈ 0.2 are in good correspondence with the experimental literature data. The normalized (on corresponding results for unfilled polymer) Young’s moduli, calculated based on experimental data for epoxy-based nanocomposites, vary from ≈1.05 to 1.15 in the presence of 1 to ≈5 wt.% of silylated montmorillonites (with various amounts of 3-Aminopropyltriethoxy silane) [20]; they are equal to ≈1.22 (at 4 wt.% of MMT modified with Octadecyl ammonium (I.30E)); to ≈1.28 (at 3.5 and 5 wt.% of MMT modified with 3-glycidoxypropyltri methoxy silane); to ≈1.53 (at 3 wt.% of MMT modified with half neutralized salt of Jeffamine ED900) [21]; to ≈1.3 (at 3.5 wt.% of MMT modified with polydopamine) [22]; and to ≈1.1 (at 3 wt.% of Nanomer I.30E organoclay and glassy state) and reach ≈2.5 and ≈4.92 (at 6 wt.% and 10 wt.% of Nanomer I.30E organoclay, respectively, and rubbery state) [23].

It can also be seen from Figure 7 that with a low degree of cross-linking with a polymer matrix the NPs with the smallest surface area have the better reinforcing effect. This is because, in this case, the system contains more LBCs than the nanocomposites with NPs having a larger surface area (see Figure 4). On the contrary, at large values of the degree of cross-linking (*RGC* > 0.2), the situation is reversed. In this case, where NPs have a large surface area, more LBCs are formed with the NP participation. As the elasticity of such chains is higher (because the stiffness of the NPs is higher than that of the matrix), this leads to a greater enhancement of the mechanical properties of the system. This effect is most noticeable for disk-shaped nanoparticles.

Thus, our model demonstrates the amplifying role of NPs as additional cross-links and the effect of their shape on the mechanical properties of nanocomposites at different degrees of cross-linking with polymer.

### 4.3. Discussion

As it has been shown above, Young’s modulus for the systems with bonded nanoparticles significantly exceeds the modulus of the unfilled matrix even at small values of the degree of their cross-linking with the matrix. This is because the NPs bonded with the matrix are directly involved in the transfer of the load through the volume of the simulation cell. They play the role of cross-linking centers, and one may, in effect, consider that the load-bearing chains go through the NPs’ volume. As NPs are rigid objects compared to the matrix, they add additional rigidity to the LBCs and, therefore, to the entire system.

Thus, the obtained results show that the load-bearing chains play a central role in forming the mechanical response of a nanocomposite based on highly cross-linked matrices. In coarse-grained models, they are chains of beads connected by springs through periodic boundary conditions. The higher density of such chains in the system leads to the higher Young’s modulus. If NPs are not bonded, they act as obstacles, which reduce the LBC density. As the repulsive short-range potential realizes intermolecular interactions in DPD, a decrease in the number of LBCs decreases the total system’s rigidity. Moreover, it was shown in [63] that varying the parameters, *a_ij_*, regulating the repulsion between NP and matrix, when the interface does not form a percolating structure, gives a negligible effect on the mechanical response of the model. Thus, the deterioration of the mechanical properties of polymers upon the introduction of nanoparticles is, in some way, an unpleasant feature of the DPD models.

It might be said that in DPD models the role of the interface in the formation of the mechanical response is not as significant as in the real nanocomposites. In fact, due to the strong interaction between the nanoparticle and the polymer near the interface, the nanocomposite often has properties completely different from those of the bulk polymer. Many experimental studies [94,95,96,97,98,99,100,101] have reported the existence of an immobilized interfacial polymer layer due to the high adhesion of the polymer chains on the surface. It has also been shown that this adhesion can be weakened by grafting polymer chains to the surface and, vice versa, enhanced by increasing the polymer-surface attraction [95,99]. These facts were investigated by coarse-grained [102,103,104,105] and atomistic [106,107,108,109,110,111,112] simulations.

As the interfacial interaction between polymers and the NP surface improves the mechanical properties of the polymer, a large surface area, in addition to the high modulus of the filler, in the molecular models should directly affect the bulk properties of the nanocomposite. In full-atomistic and coarse-grained models implemented in MD simulation, the interfacial interaction is taken into account by attractive intermolecular forces. As mentioned above, in the DPD the role of the interface is weakened due to the repulsive nature of the intermolecular forces. In our opinion, this drawback can be overcome by introducing bonds between the polymer matrix and the NP surface. The density of the number of cross-links, in this case, makes it possible to effectively take into account the strong interaction of the polymer with the matrix. We used a similar mechanism in our recent study [68]. By introducing additional bonds between beads, we made an attempt to take into account the formation of π-π stacking interactions, as they are responsible for the formation of supramolecular structures in conjugated polymers (CP). The introduction of the possibility of forming the additional bonds into the DPD model of CP allowed us, in a narrow window of parameters, to reproduce the bundles of poly-3-hexylthiophene chains. The formed scales in our model turned out to be comparable with the experimental data.

Taking into account the considerations described above, the introduction of linkers on the surface of the NP model (through which complex DPD beads can be bonded) may be interpreted in two ways. On the one hand, it simulates the presence of a cross-linking surface modifier. On the other hand, with the help of linkers, we can indirectly take into account the strong interaction between the NPs and the matrix. Thus, the introduction of additional NPs/matrix bonds allows one to improve the functionality of the DPD modeling.

## 5. Conclusions

A mesoscale model of polymer nanocomposites based on highly cross-linked polymer networks was presented. The model aims at predicting the main trends in the change in the mechanical properties of nanoparticle-filled polymers. It explicitly considers the shape of nanoparticles and the ability of the surface modifier to form chemical cross-links with a polymer matrix. For this, the NP surface (such as matrix comonomers) was constructed as composite beads from ordinary DPD beads with additional particles attached to them, called linkers. The linkers were assigned to reactive groups in the original chemical structures. New bonds can arise between linkers, which simulate the formation of chemical cross-links between the NPs and the polymer matrix. The proposed model is a significant advantage when creating multiscale simulation schemes.

This approach was used to study the mechanical properties of vitrimer systems filled with nanoparticles of different shapes. The vitrimer was based on DGEBA comonomers and tricarboxylic fatty acid. The clay nanoparticles were chosen as filler. To study the influence of the shape of the NP, we built cylindrical NPs of the same volume with different aspect ratios of diameter to height, disc-shaped, barrel-shaped, and rod-shaped. One can correlate disc-shaped and rod-shaped NPs with exfoliated layered silicates and halloysite, NPs which have the form of nanotubes. The barrel-shaped form was used as a transitional form to study the influence of the aspect ratio on the reinforcing properties of NPs.

In correspondence with the experimental data, it was shown that the introduction of nanoparticles to a polymer matrix leads to the improvement of the mechanical properties only when bonded with it. The simulations and topological analysis (performed with Khalatur’s method) reveal the mechanism underlying these effects. The main contribution to the mechanical response is the so-called load-bearing chains (LBCs), a sequence of chemically cross-linked monomers and NPs passing through the simulation cell. The introduction of NPs, having much a higher rigidity than the polymer matrix, into the LBCs causes a significant increase in the rigidity of the system as a whole. This explains the increase in the modulus of elasticity with an increase in the degree of cross-linking with the polymer matrix and the more significant contribution of NPs with a larger surface area (having a bigger aspect ratio), which can form more cross-links with the polymer matrix.

We would like to mention that the presented approach, allowing the possibility to account for chemical cross-links, can be used to study other systems with a strong interaction of the matrix with the filler (e.g., adhesion, physical cross-links, etc.). This makes it possible to expand the application areas of models with soft potentials.

## Figures and Tables

**Table 1:** The densities of load-bearing chains for the systems with *RGC* = 0.

System	$n_{x}\;[\sigma$^−2^]	$n_{y}\;[\sigma$^−2^]	$n_{z}\;[\sigma$^−2^]	Average
Unfilled network	1.67 ± 0.03	1.66 ± 0.03	1.67 ± 0.02	1.67 ± 0.03
*i*	1.08 ± 0.11	1.14 ± 0.04	1.11 ± 0.035	1.11 ± 0.03
*ii*	1.14 ± 0.07	1.17 ± 0.01	1.13 ± 0.05	1.15 ± 0.02
*iii*	1.18 ± 0.02	1.09 ± 0.07	1.08 ± 0.14	1.12 ± 0.06

## Data Availability

Data sharing is not applicable.

## Supplementary Materials

The following are available online at https://www.mdpi.com/article/10.3390/ma14216637/s1, Figure S1: Snapshots of the nanocomposites with embedded nanoparticles of the *i*, *ii* and *iii* types. (A) Examples of the initial distribution of nanoparticles. (B) Snapshots of the cross-linked nanocomposite. Linkers on the surface of nanoparticles are not shown for clarity of visualization. All colors in this figure are the same as in Figure 1. Figure S2: The orientational order parameter for axes of nanoparticles in nanocomposites before cross-linking reaction. Figure S3: Degree of conversion of the epoxy network for unfilled polymer matrix and nanocomposites with nanoparticles of three types when *f_l_* = 0 and *f_l_* = 1. Table S1: Characteristics of the substances used to parameterize the DPD model: molar volume *V*_m_, and solubility parameters δ.
