# Supplementary material for: Effect of Nanoparticles Surface Bonding and Aspect Ratio on Mechanical Properties of Highly Cross-Linked Epoxy Nanocomposites: Mesoscopic Simulations
Source: Materials (Basel). 2021 Nov 4;14(21):6637. doi: 10.3390/ma14216637 (PMC8587117; doi:10.3390/ma14216637)
Supplement: Supplementary file 1 [file materials-14-06637-s001.zip › materials-1415391-supplementary.pdf]

materials

Supplementary Materials

Effect of Nanoparticles Surface Bonding and Aspect Ratio on Mechanical Properties of Highly Cross-Linked Epoxy Nanocomposites: Mesoscopic Simulations

Maxim D. Malyshev [1], Daria V. Guseva [2], Valentina V. Vasilevskaya [2,*] and Pavel V. Komarov [1,2,*]

[1] Departments of Physical Chemistry and General Physics, Tver State University, Zhelyabova 33, 170100 Tver, Russia; bggf@bk.ru
[2] A.N. Nesmeyanov Institute of Organoelement Compounds RAS, Vavilova St. 28, 119991 Moscow, Russia; guseva@polly.phys.msu.ru
* Correspondence: vvvas@polly.phys.msu.ru (V.V.V.); pv_komarov@mail.ru (P.V.K.)

Here we present additional experimental and computer simulation data on the systems under study. All parameters are expressed in DPD units, viz., $m = 1$ (the mass of the DPD particles), $\sigma = 1$ (the unit of scale), $k_BT = 1$ (the unit of energy, T is absolute temperature, k_B is the Boltzmann constant), $(m\sigma^2/ k_BT\ T)^{1/2} = 1$ (the unit of time) [1].

S1. The Repulsion Parameters a_{ij} and the Miscibility Subsystems

The miscibility of all the species (comonomers and NPs) in DPD model are expressed through repulsion parameters a_{ij} [1] related with the Flory-Huggins parameters χ_{ij} (FH) [2,3] as follows:

$$a_{ij} = 25 + 3.497\chi_{ij}. \quad (1)$$

The smaller value of $\chi_{ij} < 0.5$, the better the miscibility of species i and j ($a_{ij} = 25$), and when $\chi_{ij} > 0.5$, the phase separation for subsystems is expected.

The well-known relationship [4,5] between χ and the Hildebrant solubility parameters δ [5–7] is used to estimate χ_{ij}:

$$\chi_{ij} = \nu(\delta_i - \delta_j)/RT + \chi_S, \quad (2)$$

where ν is the averaged molecular volume of chemical species, R is the gas constant, and χ_S is the contribution of entropy to the free mixing energy, which for many mixtures is 0.34 [8,9], and is often neglected. It should be noted that the solubility parameters are related to the cohesion energy density E_{coh}, according to the definition $\delta = (E_{coh}/V)^{1/2}$ (where V is the volume of the system). It is usually used in molecular dynamics (MD) simulations to evaluate the solubility parameters [11–13].

A semi-empirical Askadskii's method [10] provides the most rapid way to estimate the solubility parameters. For a more accurate estimation, it is necessary to use atomistic molecular dynamics simulations [11–13].

The solubility parameters were calculated for the following compounds:

1) The polymer matrix comonomers bisphenol-A epoxy resin (DGEBA) and tricarboxylic fatty acid (FAT3);

2) Surface modifiers of clays, such as (3-triethoxysilyl)-propyl-succinic anhydride (TPSA), dimethyl-dodecyl-quaternary ammonium (DDQA), methyl-dodecyl-bis-2-hydroxyethyl-quaternary ammonium (DDHQA), 3-amono-propyl-triethox-

ysilane (APTS), hexadecyl-ammonium (HDA), amino-ethyl-amino-propyl-tri-methoxy-silane (AEAPTS), 3-(glycidyloxypropyl)-trimethoxysilane (GTO) [12,14–17].

The results obtained for considered substances are placed in Table S1. The Table S1 also contains simple compounds as hydrophobic and hydrophilic molecules. As can be seen from Table S1, for DGEBA, alkanes, and water, there is an acceptable agreement between Askadskii's method, MD, and experimental results. The acceptable agreement is also observed for Askadskii's method with the experiment in the case of surface modifier DDQA.

Table S1. Characteristics of the substances used to parameterize the DPD model: molar volume V_m, and solubility parameters δ.

	Ascadskii		MD*	Literature data
	V_m [cm^3/Mol]	δ [MPa$^{1/2}$]	δ [MPa$^{1/2}$]	δ [MPa$^{1/2}$]
Matrix comonomers				
DGEBA	333	20.3	20.5±0.1	17–27 [18], 21.9 [19]
FAT3	956	19.6		
Surface modification of clays				
TPSA	304	18.2		
DDQA	383	16.4		17.9 [24]
DDHQA	446	19.8		
APTS	199	17.5		
HDA	327	17.6		
AEAPTS	222	17.9		
GTO	236	17.2		
Reference molecules				
C_6H_{14}	125	14.5	14.7±0.1	14.90[5], 14.8[20]
C_8H_{18}	163	15.5	15.0±0.2	15.5[5], 14.0[20]
H_2O	17.7	40.9	47.1 [11]	47.9 [21], 47 [22,23]

*) The calculations were performed using the molecular dynamics package LAMMPS [25], second-generation polymer-consistent force field (PCFF) [26–28], and the simulation protocol that we developed earlier [11].

The results in the Table S1 indicate that the comonomers DGEBA (has aromatic rings) and FAT3 (has long alkyl chains) are non-polar ($\chi_{DGEBA,C8H18} = 2.3$, $\chi_{DGEBA,H2O} = 29.8$, $\chi_{DGEBA,C8H18} = 3.8$, $\chi_{DGEBA,H2O} = 88.6$). The same conclusion can be made about the selected surface modifiers due to long alkyl chains in their structures (for example $\chi_{TPSA,C8H18} = 0.68$, $\chi_{TPSA,H2O} = 33.2$, $\chi_{TPSA,C8H18} = 0.23$, $\chi_{TPSA,H2O} = 28.5$).

Estimation of the Flory Huggins parameters using formula (2) for the pairs DGEBA-surface modifier and FAT3-surface modifier gives, respectively, the following ranges of values 0.04-2.18 and 0.01-2.75. The results obtained indicate that by appropriate selection of the surface modifier, it is possible to achieve good compatibility of clay nanoparticles with the polymer.

S2. Supplementary Figures for Paragraph of System Preparation

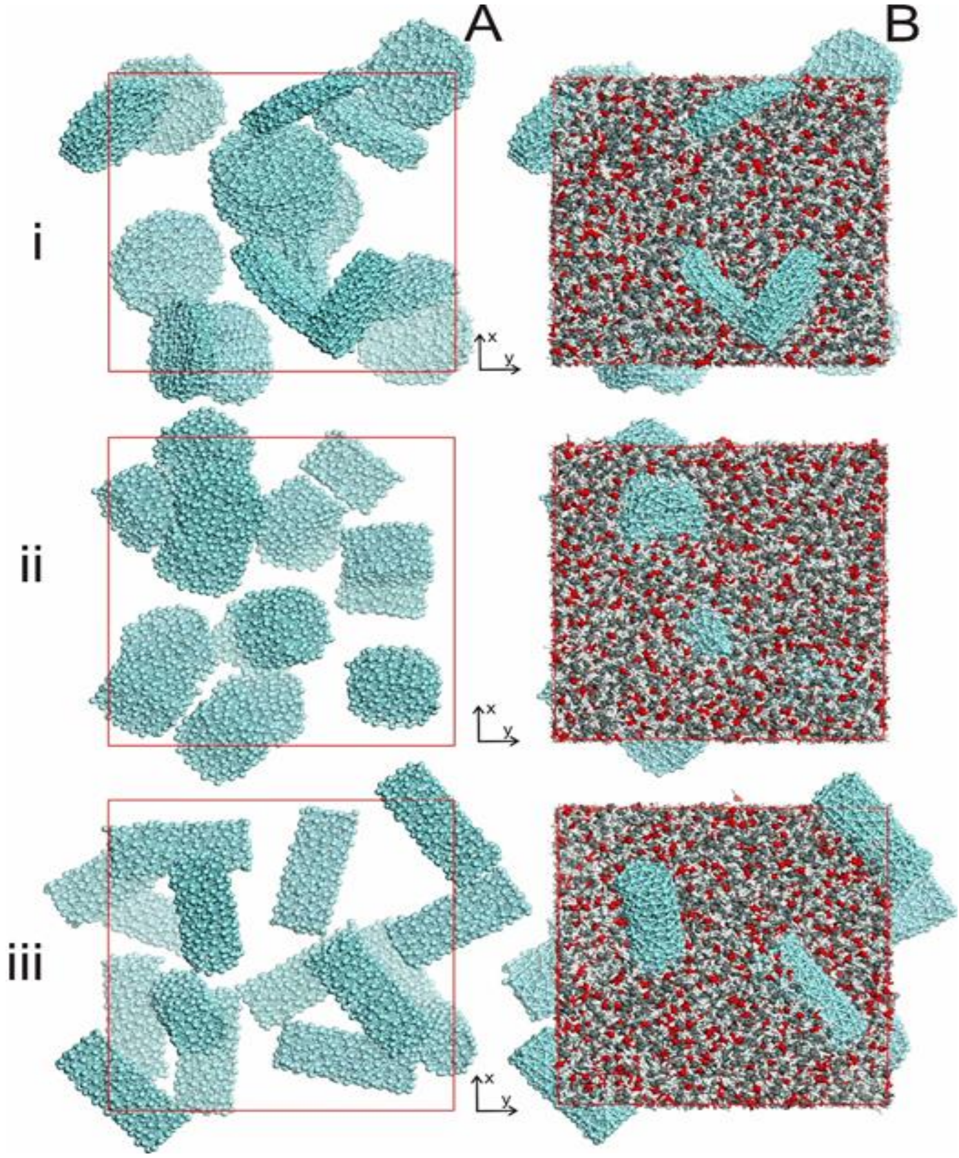

Figure S1. Snapshots of the nanocomposites with embedded nanoparticles of the *i*, *ii* and *iii* types. A) Examples of the initial distribution of nanoparticles. B) Snapshots of the cross-linked nanocomposite. Linkers on the surface of nanoparticles are not shown for clarity of visualization. All colors in this figure are the same as in Figure 1.

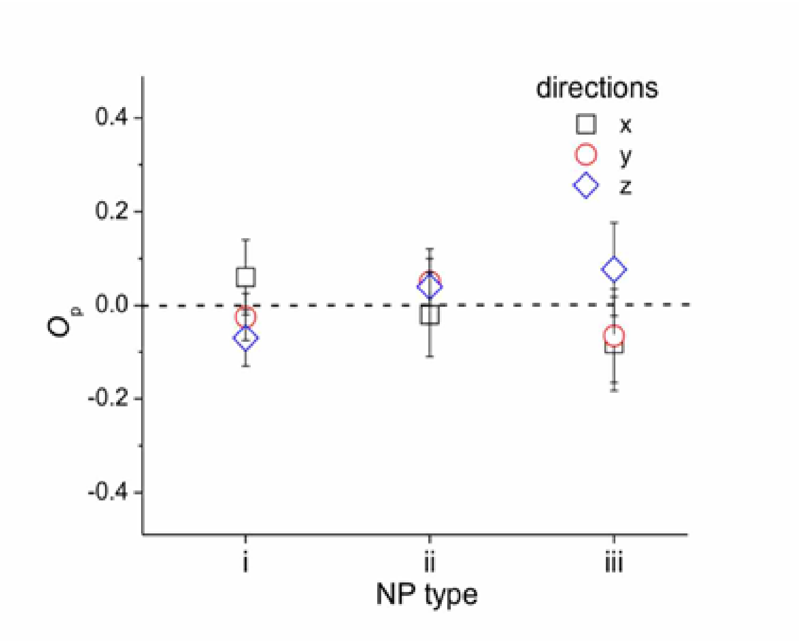

Figure S2. The orientational order parameter for axes of nanoparticles in nanocomposites before cross-linking reaction.

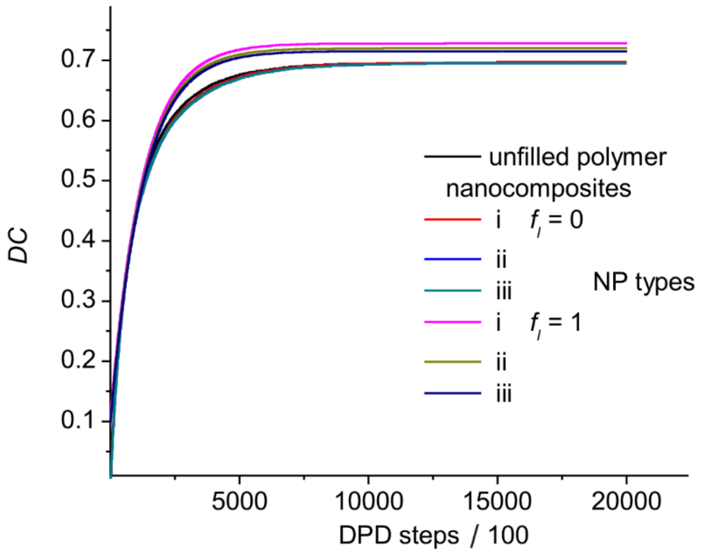

Figure S3. Degree of conversion of the epoxy network for unfilled polymer matrix and nanocomposites with nanoparticles of three types when $f_l = 0$ and $f_l = 1$.

References

1. R. D. Groot and P. B. Warren, The Journal of Chemical Physics, 1997, 107, 4423–4435.
2. P. J. Flory, J. Chem. Phys., 1941, 9, 660–661.
3. M. L. Huggins, J. Chem. Phys., 1941, 9, 440–440.
4. X. D. Guo, L. J. Zhang, Z. M. Wu and Y. Qian, Macromolecules, 2010, 43, 7839–7844.
5. C. M. Hansen, Hansen Solubility Parameters: A User's Handbook, Second Edition, Taylor Francis, 2007.
6. L. I. Stiel, AIChE Journal, 1971, 17, iv–iv.
7. C. Reichardt, Solvents and Solvent Effects in Organic Chemistry: Edition 3, John Wiley Sons, 2006.
8. E. Spohr, P. Commer and K. A. A., J. Phys. Chem. B., 2002, 106, 10560–10569.
9. M. W. Verbrugge, R. F. Hill, Schneider and E. W., AIChE Journal, 1992, 38, 93–100.
10. A. A. Askadskii, Computational Materials Science of Polymers, Cambridge Int Science Publishing, 2011.
11. P. V. Komarov, I. N. Veselov and P. G. Khalatur, Polymer Science Series A, 2010, 52, 191–208.

12. A. A. Markina, V. A. Ivanov, P. V. Komarov, Chemical Physics Letters 2016, 653, 24–29.
13. V.Y. Rudyak, A.A. Gavrilov, D.V.Guseva, S.-H. Tung, P.V. Komarov, Mol. Syst. Des. Eng. 2020, 5, 1137–1146.
14. K. Zhang, L. Wang, F. Wang, G. Wang, Z. Li, J. Appl. Polym. Sci. 2004, 91, 2649–2652.
15. S.R. Ha, S.H. Ryu, S.J. Park, K.Y. Rhee, Mater. Sci. Eng. A Struct. Mater. 2007, 448, 264–268.
16. S.S. Ray, Macromol. Chem. Phys. 2014, 215, 1162–1179.
17. S.-L. Bee, M.A.A. Abdullah, S.-T. Bee, L.T. Sin, A.R. Rahmat, A Review. Prog. Polym. Sci. 2018, 85, 57–82.
18. https://polymerdatabase.com/polymers/bisphenol-adiglycidyletherepoxyresin.html
19. R. Wang, T. P. Schuman, eXPRESS Polymer Letters 2013, 7, 272–292.
20. G. Wypych, Handbook of Solvents, ChemTec Publishing, 2001.
21. Wescott J.T., Qia Y., Subramanian L., Capehart, T.W. // J. Chem. Phys. 2006. V.124. P.134702.
22. "Polymer Handbook", edited by J. Brandrup, E. H. Immergut, and E. A. Grulke New York :Wiley. US. 2003.
23. D.L. Ho, C.J. Glinka, Chem Mater 2003, 15,1309–12.
24. A. Choudhury, A. K. Bhowmick, C. Ong, Polymer 2009, 50, 201–210.
25. S. Plimpton, Journal of Computational Physics, 1995, 117, 1–19.
26. H. Sun, S. J. Mumby, J. R. Maple and A. T. Hagler, J. Am. Chem. Soc., 1994, 116, 2978–2987.
27. H. Sun, Macromolecules, 1995, 28, 701–712.
28. H. Sun, S. Mumby, J. Maple and A. Hagler, J. Phys. Chem., 1995, 99, 5873–5882.
